# Glycyrrhizin Alleviates the Damage Caused by Zearalenone and Protects the Glandular Stomach of Chickens

**DOI:** 10.3390/ani15040489

**Published:** 2025-02-09

**Authors:** Tong Sun, Fuhan Wang, Man Qian, Jingjing Wang, Mengyao Guo

**Affiliations:** College of Veterinary Medicine, Northeast Agricultural University, Harbin 150030, China; suntong@neau.edu.cn (T.S.); wangfuhan1997@sina.com (F.W.); qm18437916979@163.com (M.Q.); s230602012@neau.edu.cn (J.W.)

**Keywords:** zearalenone, glycyrrhizic acid, inflammation, oxidative stress, apoptosis, necrosis

## Abstract

Zearalenone (ZEA) is a kind of mycotoxin that widely contaminates food and feed and poses a threat to poultry farming. As a natural extract, glycyrrhizin acid (GA) has antioxidant, antibacterial, and anti-inflammatory effects. Although studies have revealed the toxic effects of ZEA on the liver, the mechanism by which GA reduces ZEA’s toxic protective glandular stomach remains unclear. In order to study the therapeutic effect of GA on tissue damage caused by ZEA, we conducted in vivo and in vitro experiments to compare the expression of inflammation, oxidative stress, apoptosis, and necrosis. The results showed that ZEA can induce inflammation in tissues and cells, inducing apoptosis and necrosis. In addition, GA can alleviate the toxic effects caused by ZEA and protect cells. Dietary GA significantly increased the antioxidant capacity of glandulae and inhibited the overexpression of NFκB/IκB-α and its mediated inflammatory response. Moreover, GA decreased the expression of pro-apoptotic factors and necrosis factors, thereby alleviating apoptosis and necrosis of chicken glandular stomach cells. At present, the mechanism of ZEA damage to livers and lungs has been confirmed by studies. However, there have been no studies on GA alleviating the damage caused by ZEA to the glandular stomach. Therefore, the purpose of this study was to explore the mechanism of GA alleviating the damage caused by ZEA in the glandular stomach through in vivo and in vitro experimental comparison. The results may provide some reference for the solution of feed contamination.

## Simple Summary

Zearalenone (ZEA) is a mycotoxin that widely contaminates feed. Glycyrrhizin acid (GA) is a traditional Chinese medicine extract of licorice. This study found that GA could alleviate the damage to chicken glands and stomachs caused by ZEA in vivo, reduce the levels of oxidative stress in the body, and reduce the number of instances of apoptosis and programmed necrosis. GA may inhibit apoptosis and programmed necrosis mediated by inflammation through the NFκB pathway and protect the glandular gastric tissue. These results provide indirection for toxicological studies of ZEA and therapeutic effects of GA and help further explore the toxic effects of mycotoxins on the digestive tract.

## 1. Introduction

Zearalenone, a mycotoxin [1,2], widely pollutes various grains around the world, such as wheat, barley, corn, sorghum, rye, rice, and other grains and feeds [3]. ZEA causes varying degrees of harm to humans and animals [4] by changing the morphology and function of tissues and organs. The toxic effects of ZEA can also be aggravated in combination with other toxins [5]. ZEA impairs intestinal integrity and function, leading to pathological symptoms and tissue damage [6]. ZEA is widely absorbed by mice, pigs, poultry, and humans through the oral consumption of contaminated grains, foods, and feeds. Studies have shown that ZEA is rapidly absorbed through the gastrointestinal tract after oral ingestion. Feed contamination has been shown to cause intestinal inflammation and cecum injury in pigs [7,8], and a ZEA-contaminated diet was shown to reduce rumen PH and impair their function [9,10]. ZEA is widely distributed in various tissues and organs of animals and is slowly metabolized to clear the body. In addition, ZEA can be deposited in animals or animal products through feed and continue to cause harm [11].

The nutritional value of feed contaminated with mycotoxin is greatly reduced, which affects animal health, can cause tissue inflammation, and reduces antioxidant enzyme activity. ZEA, as an estrogen toxoid, affects the expression of inflammatory factors in poultry serum and ROS expression in vivo [12,13]. Studies have shown that ZEA induces apoptosis and inflammation of jejunal epithelial cells, leading to jejunal tissue damage [14]. Feeding low-dose ZEA-contaminated feed for a long time can cause intestinal inflammation in piglets. Diets contaminated with a high dose of ZEA can affect the antioxidant capacity of IPEC-J2 cells in the small intestine of pigs, resulting in ROS accumulation and mitochondrial apoptosis [15]. ZEA can induce hepatocyte apoptosis and affect liver function by activating the NFκB-signaling pathway by regulating inflammation-related factors [16,17,18]. Feeding chickens with different levels of ZEA contamination, we found that ZEA exposure significantly increased malondialdehyde (MDA) levels and inhibited catalase (CAT) activity, suggesting that ZEA induces tissue damage in chicks by regulating oxidative stress [19]. The results show that T-2 toxin can significantly affect the activity of digestive enzymes, causing intestinal mucosal inflammation, necrosis, and other symptoms. It enters the gastrointestinal tract through the mouth and begins to regulate genes involved in oxidative stress, inflammation, apoptosis, cancer, and other related genes, resulting in glandular and gastric damage [20].

At present, no specific medicine exists for damage caused to poultry by feed contamination by ZEA. Licorice is derived from the roots and rhizomes of the licorice plant and shows good antioxidant, antifungal, and anti-inflammatory effects, making it a widely developed Chinese medicine therapeutic agent. Glycyrrhizin, a compound extracted from licorice root, is one of licorice’s most potent therapeutic ingredients [21,22]. GA mainly inhibits the expression of the nuclear factor kappa B (NFκB) pathway and then inhibits the encoding of important genes such as inflammatory cytokines, anti-apoptotic factors, and anti-necrosis factors so as to achieve anti-inflammatory effects. To date, no studies have been conducted on GA alleviating the damage caused by ZEA to the body. Therefore, this study mainly describes the mechanism of ZEA damage to the chicken glandular stomach and how GA alleviates panapoptosis caused by ZEA in the body, providing a certain reference for a solution to feed contamination.

## 2. Materials and Methods

### 2.1. Animals and Groups

Animal experiments were conducted in accordance with the guidelines of the Animal Care and Use Committee of Northeast Agricultural University. Fifty 7-day healthy chickens were randomly divided into five groups: the control group (Con group), the ZEA group, the ZEA+LGA group, the ZEA+MGA group, and the ZEA+HGA group. Each chicken is kept in a separate cage. The control group (Con) was fed common chicken feed and water. Chicken feed is bought commercially. It is composed of corn, soybean meal, bran, calcium bicarbonate, and sodium chloride. The feed does not contain fungal inhibitors. The feed purchased is verified by the kit to be ZEA-free. The ZEA group was fed 2 mg/kg of ZEA and ordinary chicken feed every 24 h. ZEA and GA were acquired from Shanghai Aladdin Biochemical Technology Co., LTD (Shanghai, China). The ZEA+LGA group was provided 2 mg/kg of ZEA every 24 h, and the chickens were fed diets containing 50 mg/kg of GA. The ZEA+MGA group was provided 2 mg/kg of ZEA every 24 h and fed chicken feed with 100 mg/kg of GA content. The ZEA+HGA group was provided 2 mg/kg of ZEA every 24 h, and the chickens were fed diets containing 150 mg/kg of GA. ZEA is mixed with feed. The stability of ZEA intake is ensured by maintaining the daily feed intake of each chicken. All the chickens were allowed free access to food and water and were kept at room temperature. After 21 days of exposure to ZEA, all animals were anesthetized and killed. We collected glandular gastric tissue. Part of the fresh glandular gastric tissue was fixed using paraformaldehyde. Other glandular gastric tissues were quickly cryogenically preserved in liquid nitrogen for subsequent analysis.

### 2.2. Histopathology Staining

The glandular stomach tissue was fixed with 4% paraformaldehyde, and 4 μm sections were prepared by embedding the tissue with paraffin wax. After dewaxing the sections, they were stained with hematoxylin for 5 min, rinsed with eosin for 3 min, and sealed with neutral glue after dehydration. The slices were then observed under a microscope (digital tissue section scanner, 3DHISTECH, Budapest, Hungary), and stained images were obtained using an image acquisition system (CaseViewer2.4, 3DHISTECH, Budapest, Hungary).

### 2.3. Cell Isolation Culture

Several chicken embryo glandular stomachs were isolated under sterile condition, and 0.25% trypsin was digested at 37 °C for 10 min. Digestion was terminated with DMEM (HyCyte, Suzhou, Jiangsu, China) containing 10% fetal bovine serum (FBS, HyCyte, Suzhou, Jiangsu, China). Use 0.1% type II collagenase for digestion for another 10 min. Then, DMEM containing 10% FBS was added to terminate digestion. Finally, the cells were resuspended in DMEM containing 10% FBS and uniformly inoculated into T25 cell vials. The new paper was placed horizontally in an incubator at 37 °C and 5%CO_2_ for culture. Then, 0.25% trypsin and 0.1% type II collagenase were purchased from Shanghai Biyuntian Biotechnology Co., Ltd. (Shanghai, China). Control group (Con): the glandular gastric epithelial cells were cultured normally. ZEA group: the cells were cultured with 2 μM of ZEA. ZEA and low-dose GA co-treatment group (ZEA+LGA): the cells were cultured with 2 μM ZEA and 50 μM GA. ZEA and medium-dose GA co-treatment group (ZEA+MGA): the cells were cultured with 2 μM of ZEA and 100 μM of GA. ZEA and high-dose GA co-treatment group (ZEA+HGA): the cells were cultured with 2 μM of ZEA and 150 μM of GA.

### 2.4. TUNEL Analysis

The paraffin sections were dewaxed twice for 10 min, before and after, and then hydrated with a high-to-low concentration of ethanol solution. ProteinaseK working fluid was used to permeate sections to promote TUNEL working fluid penetration into the cells [23]. Finally, TUNEL working solution was used to observe the apoptosis of cells in the tissues under a fluorescence microscope.

### 2.5. Reactive Oxygen Species (ROS) Detection

The cells were cultured in groups, washed with PBS, and diluted. Then, a DCFH-DA probe was added, and the cells were incubated at 37 °C away from light for 30 min and then washed with PBS after discarding the solution. Fluorescence intensity was observed under a fluorescence microscope [24]. The ROS kit was acquired from Nanjing Jiancheng Biotechnology Co. (Nanjing, China).

### 2.6. Antioxidant Enzyme Detection

A total of 0.1 g of chicken glandular stomach tissue was weighed, 1 mL of normal saline was added, the mixture was ground to a homogenate at 4 °C and centrifuged at 4000 rpm at 4 °C for 15 min, and the supernatant was obtained. A total of 1 mL of PBS-suspended cells was then ground at 4 °C and centrifuged at 4000 rpm for 15 min to obtain a supernatant. MDA content was determined via the TBA method, SOD activity was determined via the hydroxylamine method, CAT activity was determined via the ammonium molybdate method, T-AOC activity was determined via the colorimetric method, and GSH-PX activity was determined via the enzymatic reaction method. All procedures followed the instructions of the operations conducted at Nanjing Jiancheng Co., Ltd., Nanjing, China.

### 2.7. ELISA

An ELISA kit (Meimian, Yancheng, Jiangsu, China) was used to detect the production of inflammatory factors IL-6, IL-10, IL-1β, and TNF-α in chicken glandular stomach tissue; 0.1g of tissue was weighed and ground with 1 mL of pre-cooled PBS; cells were collected and suspended with PBS; and the tissue and cells were thoroughly ground with a Tissue Lyser. Centrifugation took place at 4000 rpm for 15 min. Then, the kit instructions were followed for the test. OD values were measured at 450 nm using an enzyme-labeled instrument. The protein concentration of each factor in the sample was calculated by drawing a standard curve.

### 2.8. qRT-PCR

The mRNA expression level was detected via qRT-PCR. RNA from chicken glandular stomach tissues and cells was extracted using Trizol (Invitrogen, Carlsbad, CA, USA), and reverse transcription was conducted with the first-strand cDNA synthesis kit (Bioer Technology, Hangzhou, China). The target genes were amplified with a 20 μL system (1 μL of cDNA, 10 μL of 2×SYBR Green PCR premix, 3.4 μL of water without RNase, 0.4 μL of a forward primer, and 0.4 μL of a reverse primer), and the total number of cycles was 40. Subsequently, qPCR analysis was conducted on a Light Cycler@480 System (Roche, Basel, Switzerland) following the BioEasy Master Mix kit instructions. With GAPDH as the internal reference gene, the calculated result is expressed as 2^−ΔΔCt^, and the primers used in this experiment are shown in Table 1.

### 2.9. Western Blotting

Protein was extracted from glandular stomach tissue and cells and then ground. The concentration of histamine was determined and adjusted following the BCA (Beyotime, Shanghai, China) method. The supernatant was obtained from chicken gastric epithelial cells via ice lysis for 30 min and centrifugation at 4 °C 12,000 rpm for 10 min. The cell protein concentration was measured following the BCA method and then allowed to level out. The protein samples were separated via 12% SDS-PAGE and transferred to an NC membrane (Sigma, Mannheim, Germany). After 5% skim milk was sealed for 2 h, the NC film was placed in the primary antibody and incubated at 4 °C overnight. After TBST cleaning, the membrane was incubated in the secondary antibody for 1 h away from light. The image was exposed and retained with ECL luminescent solution (Gibco, Baltimore, ML, USA) and analyzed with ImageJ (National Institutes of Health, Bethesda, MD, USA) [25].

### 2.10. AO/EB

The apoptosis and necrosis of chicken glandular gastric epithelial cells were detected via the AO/EB method. AO and EB were added 1:1 to 1mL of PBS, and 200 μL were added to each well of the cell plate and stained in the dark for 10 min. Images were obtained by fluorescence microscopy [26].

### 2.11. Statistical Analysis

All data are presented as standard deviation (SD) ± mean, and at least three biological replicates were performed for all experiments. Group differences were analyzed by one-way analysis of variance (ANOVA) using GraphPad Prism software 9.0. “*” indicates that a *p*-value < 0.05 is considered significant.

## 3. Results

### 3.1. GA Alleviates ZEA-Induced Tissue Damage and Oxidative Stress

**Figure 1:**
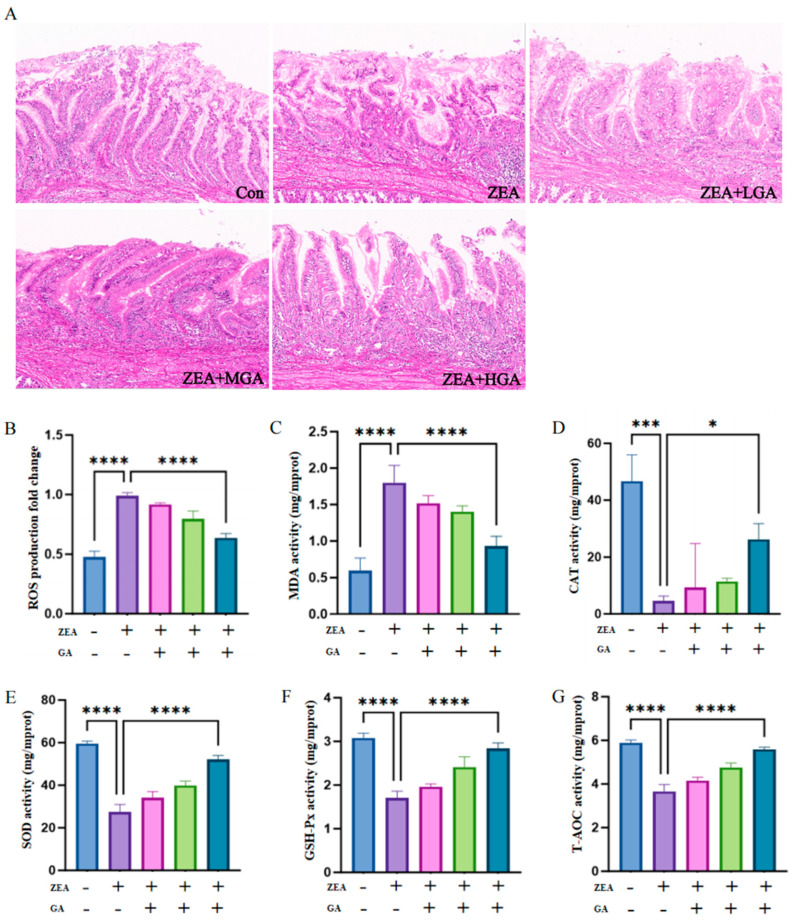
GA alleviates the effects of histological damage and oxidative stress caused by ZEA. (**A**) HE staining of chicken glandular stomach sections in the control group and at different GA concentrations. (**B−G**) Effects of control group and different concentrations of GA on oxidative stress in glandular gastric tissue of chickens (*n* = 10). * Means with different superscripts within the same column differ (*p* < 0.05). *** Means with different superscripts within the same column differ (*p* < 0.005). **** Means with different superscripts within the same column differ (*p* < 0.001).

A histological examination with HE staining (Figure 1A) showed that the glandular gastric villi were arranged regularly and that the glandular gastric structure was complete in the Con group. In contrast, the ZEA group showed villi breakage and the presence of many inflammatory cells. GA gradually restored villi arrangement and structural integrity in a dose-dependent manner, but a small amount of inflammatory infiltration and villi breakage still occurred. In order to investigate whether GA can participate in oxidative stress and reduce the effect of ZEA on chicken glandular stomach, ROS and antioxidant enzyme activities in chicken glandular stomach were detected in this experiment. ZEA caused a significant increase in ROS levels, while the ZEA-induced ROS levels decreased after GA intervention (*p* < 0.001) (Figure 1B). MDA is a marker of lipid peroxidation, and the production of MDA significantly increased under the effects of ZEA, while the level of MDA significantly decreased under the effects of GA (*p* < 0.001) (Figure 1C). With the increase in GA dose, the activities of CAT, SOD, GSH-PX, and T-AOC increased significantly, while ZEA decreased the activities of the above antioxidant enzymes (Figure 1D–G).

### 3.2. GA Relieved ZEA-Induced Interstitial Inflammation

**Figure 2:**
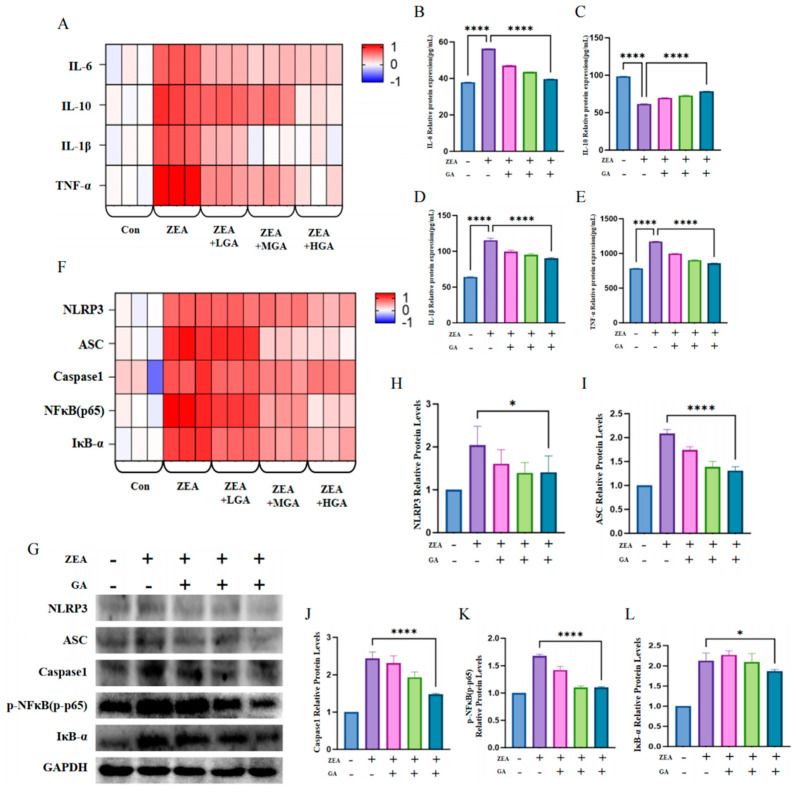
Effects of ZEA exposure and GA on glandular gastric inflammation and the NFκB/IκB−α−signaling pathway in chickens. (**A**) mRNA levels of *IL-6*, *IL-10*, *IL-1β*, and *TNF-α* (*n* = 3). (**B**) Protein expression of IL−6 (*n* = 10). (**C**) Expression of IL−10 protein (*n* = 10). (**D**) Protein expression of IL−1β (*n* = 10). (**E**) Protein expression of TNF-α (*n* = 10). (**F**) mRNA levels of *NLRP3*, *ASC*, *Caspase1*, *NFκB p65*, and *IκB−α* (*n* = 3). (**G**) Western blot images of proteins associated with the NFκB/IκB−α−signaling pathway (*n* = 10). (**H**) NLRP3 protein expression (*n* = 10). (**I**) Protein expression of ASC (*n* = 10). (**J**) Protein expression of Caspase1 (*n* = 10). (**K**) Protein expression of p−NFκB (*n* = 10). (**L**) Protein expression of IκB−α (*n* = 10). * Means with different superscripts within the same column differ (*p* < 0.05). **** Means with different superscripts within the same column differ (*p* < 0.001).

In order to investigate the mechanism of the inflammatory response induced by ZEA in the chicken glandular stomach and the alleviating effect of GA, the expression of the inflammatory cytokine *IL-6*, *IL-10*, *IL-1β*, and *TNF-α* genes was detected (Figure 2A). After exposure to ZEA, the mRNA levels of *IL-6*, *IL-1β*, and *TNF-α* increased, while the expression of *IL-10* mRNA decreased. Furthermore, the mRNA levels of inflammatory factors *IL-6*, *IL-1β*, and *TNF-α* decreased and the mRNA levels of *IL-10* increased after the application of GA. At the protein level, the expression of inflammatory cytokines was consistent with the trend for their mRNA levels. In the ZEA group, the expression of IL-6, IL-1β, and TNF-α significantly increased, while the expression of IL-10 protein decreased. After GA intervention, the expression of IL-6, IL-1β, and TNF-α significantly decreased, while the expression of IL-10 significantly increased (*p* < 0.001) (Figure 2B–E). In order to further examine whether the NFκB/IκB-α-signaling pathway participates in the mechanism behind how GA alleviates glandular gastric inflammation in chickens, the expression of the signaling pathway genes and proteins was detected. ZEA exposure significantly increased the mRNA levels of *NLRP3*, *ASC*, *Caspase1*, *NFκB p65*, and *IκB-α*. However, the mRNA levels of *NLRP3*, *ASC*, *Caspase1*, *NFκB p65*, and *IκB-α* decreased when GA was added to the feed (Figure 2F). The protein expression of the NFκB/IκB-α-signaling pathway was similar to its mRNA expression. Compared with the ZEA exposure group, the group in which GA was added significantly reduced the protein expression of NLRP3, ASC, Caspase1, p-NFκB p65, and IκB-α (*p* < 0.05) (Figure 2H–L).

### 3.3. GA Reduced ZEA-Induced Histiocytic Death

**Figure 3:**
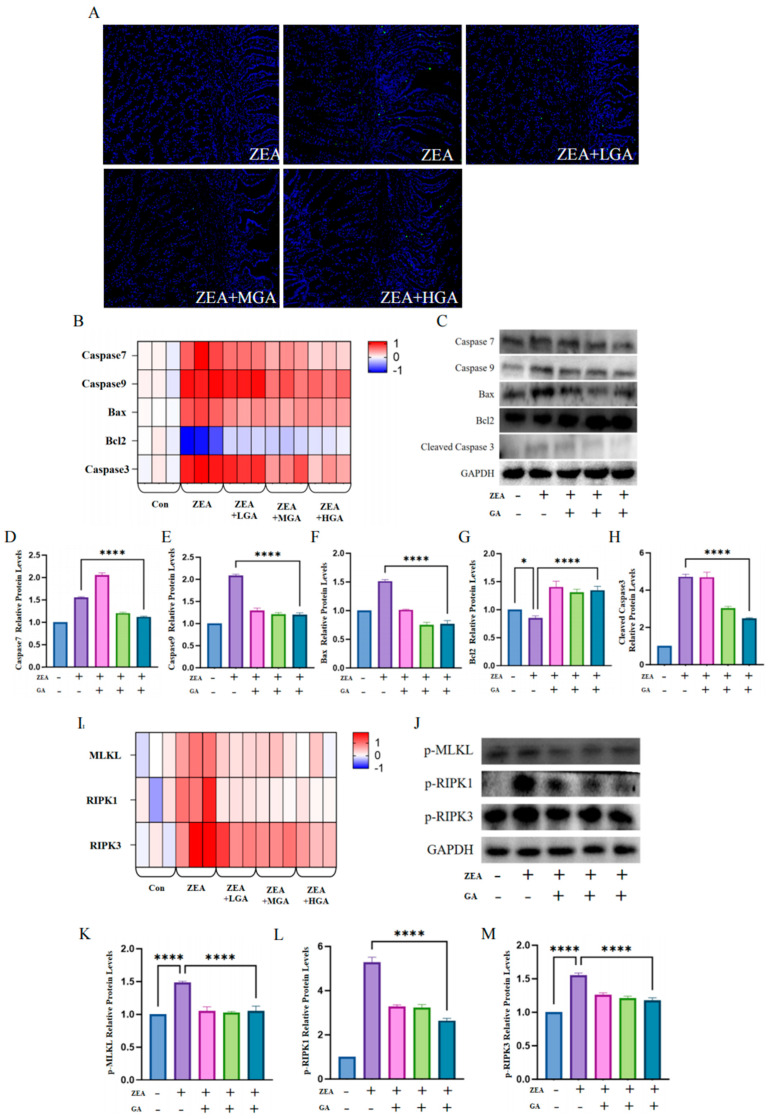
Effect of GA intervention on ZEA−induced cell death. (**A**) TUNEL−positive cells are stained green. Scale bar = 50. (**B**) mRNA levels of *Caspase7*, *Caspase9*, *Bax*, *Bcl2*, and *Caspase3* (*n* = 3). (**C**) Protein immunoblot images of Caspase7, Caspase9, Bax, Bcl2, and Caspase3 (*n* = 10). (**D**) Protein expression of Caspase7 (*n* = 10). (**E**) Protein expression of Caspase9 (*n* = 10). (**F**) Protein expression of Bax (*n* = 10). (**G**) Protein expression of Bcl2 (*n* = 10). (**H**) Cleaved Caspase3 protein expression (*n* = 10). (**I**) mRNA levels of *RIPK1*, *RIPK3*, and *MLKL* (*n* = 3). (**J**) Western blot images of p−MLKL, p−RIPK1, and p−RIPK3 (*n* = 10). (**K**) Protein expression of p−MLKL (*n* = 10). (**L**) Expression of p−RIPK1 protein (*n* = 10). (**M**) Protein expression of p−RIPK3 (*n* = 10). * Means with different superscripts within the same column differ (*p* < 0.05). **** Means with different superscripts within the same column differ (*p* < 0.001).

TUNEL staining was used to detect cell apoptosis in the gizzard tissue of chickens. Compared with the control group, the relative fluorescence intensity of TUNEL-positive cells in the ZEA group significantly increased. Compared with the ZEA group, the relative fluorescence intensity of TUNEL-positive cells in the GA group was lower and dose-dependent (Figure 3A). Further detection of the apoptosis-related factors showed that ZEA significantly promoted the mRNA expression of pro-apoptotic factors *Caspase7*, *Caspase9*, *Bax*, and *Caspase3* and inhibited the mRNA expression of anti-apoptotic factor *Bcl2*. GA decreased the mRNA expression of pro-apoptotic factors *Caspase7*, *Caspase9*, *Bax*, and *Caspase3* and increased the mRNA expression of anti-apoptotic factor *Bcl2* (Figure 3B). The expression levels of the apoptosis-related factor proteins were basically consistent with their mRNA expression levels (Figure 3C–H). In addition, ZEA exposure increased the mRNA expression levels of cell necrosis regulators *RIPK1*, *RIPK3*, and *MLKL*, while the use of GA decreased the mRNA expression levels of *RIPK1*, *RIPK3*, and *MLKL* (Figure 3L). Furthermore, the expression of the p-MLKL, p-RIPK1, and p-RIPK3 proteins was detected (Figure 3J). Notably, the phosphorylated expression of RIPK1, RIPK3, and MLKL plays an important role in this process. A phosphorylated protein analysis showed that ZEA significantly upregulated the expression levels of p-RIPK1, p-RIPK3, and p-MLKL, and the addition of GA in the diet decreased the expression levels of p-RIPK1, p-RIPK3, and p-MLKL (*p* < 0.001) (Figure 3K–M).

### 3.4. GA Inhibits ZEA-Induced Cellular Oxidative Stress

**Figure 4:**
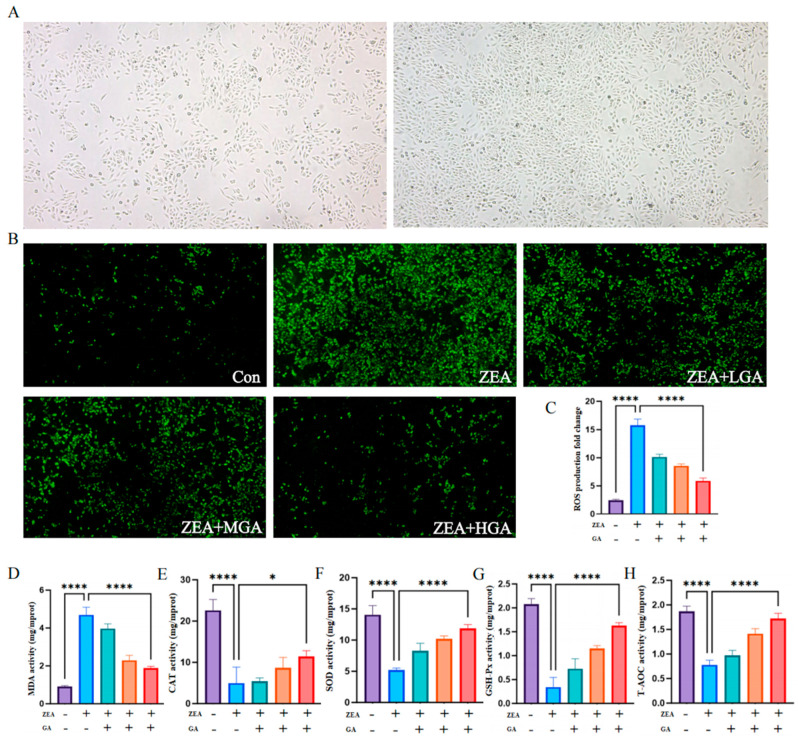
Effects of GA on ZEA−induced oxidative stress on chicken gastric gland epithelial cells. (**A**) Isolation and culture of chicken glandular gastric epithelial cells. (**B**) ROS staining of chicken glandular gastric epithelial cells under different concentrations of GA. (**C−H**) Effects of control group and different concentrations of GA on oxidative stress of chicken glandular gastric epithelial cells (*n* = 10). * Means with different superscripts within the same column differ (*p* < 0.05). **** Means with different superscripts within the same column differ (*p* < 0.001).

In order to investigate whether GA inhibits oxidative stress and protects chicken glandular gastric epithelial cells, primary cells were first extracted from chicken embryos for passage culture (Figure 4A). Fluorescence was used to detect the expression of reactive oxygen species (ROS) in chicken glandular gastric epithelial cells. Compared with the control group, the ROS level significantly increased after ZEA treatment, and the level of ROS decreased with GA intervention (Figure 4B). MDA is a marker of lipid peroxidation. Under the action of ZEA, the production of MDA significantly increased, while under the action of GA, the level of MDA significantly decreased (*p* < 0.001) (Figure 4C). Furthermore, ZEA decreased the levels of catalase (CAT), total superoxide dismutase (SOD), and GAutathione peroxidase (GSH-PX) and the total antioxidant capacity (T-AOC), and the intervention of GA increased the expression levels of CAT, SOD, GSH-PX, and T-AOC in a dose-dependent way (*p* < 0.05) (Figure 4D–H).

### 3.5. GA Protects Cells by Alleviating ZEA-Induced Inflammation Through the Nκb Pathway

**Figure 5:**
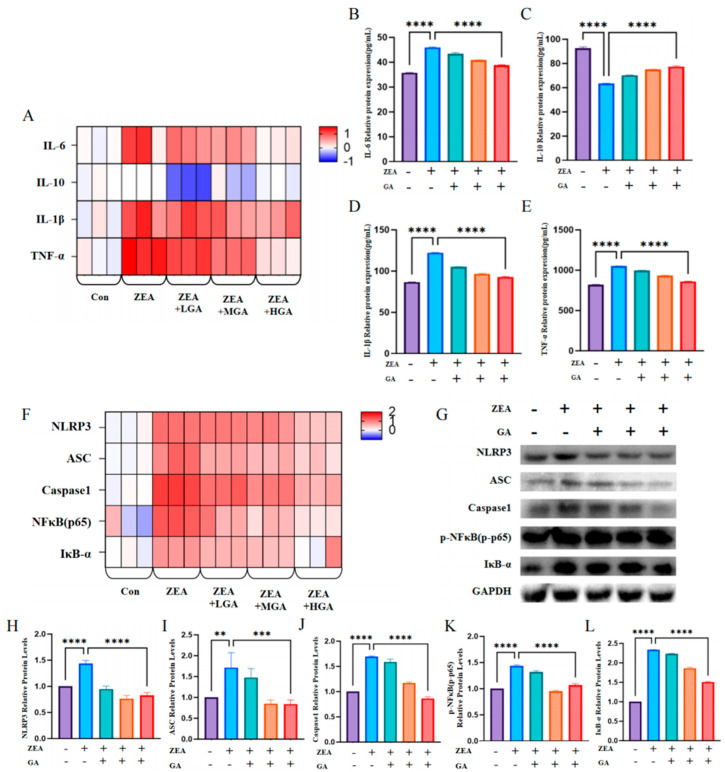
Effects of ZEA exposure and GA on inflammation and NFκB/IκB−α−signaling pathways in chicken Glandular gastric epithelial cells. (**A**) mRNA levels of *IL−6*, *IL−10*, *IL−1β*, and *TNF-α* (*n* = 3). (**B**) Protein expression of IL−6 (*n* = 10). (**C**) Expression of IL−10 protein (*n* = 10). (**D**) Protein expression of IL−1β (*n* = 10). (**E**) Protein expression of TNF-α (*n* = 10). (**F**) mRNA levels of *NLRP3*, *ASC*, *Caspase1*, *NFκB p65*, and *IκB−α* (*n* = 3). (**G**) Western blot images of proteins associated with the NFκB/IκB−α−signaling pathway (*n* = 10). (**H**) NLRP3 protein expression (*n* = 10). (**I**) Protein expression of ASC (*n* = 10). (**J**) Protein expression of Caspase1 (*n* = 10). (**K**) Protein expression of p−NFκB (*n* = 10). (**L**) Protein expression of IκB−α (*n* = 10). ** Means with different superscripts within the same column differ (*p* < 0.01). *** Means with different superscripts within the same column differ (*p* < 0.005). **** Means with different superscripts within the same column differ (*p* < 0.001).

In order to investigate the mechanism of inflammation induced by ZEA in chicken glandular stomach epithelial cells and the alleviating effect of GA, the expression of the inflammatory cytokine *IL-6*, *IL-10*, *IL-1β*, and *TNF-α* genes was detected (Figure 5A). After cell exposure to ZEA, the *IL-6*, *IL-1β*, and *TNF-α* mRNA levels increased, and *IL-10* mRNA expression decreased. After the addition of GA, the mRNA levels of inflammatory factors *IL-6*, *IL-1β*, and *TNF-α* decreased, and the expression level of *IL-10* increased. The protein results obtained via ELISA were consistent with the mRNA results (Figure 5B–E). To further verify the role of the NFκB/IκB-α-signaling pathway in the cells, the expression of related genes and proteins was detected. ZEA exposure significantly increased the mRNA levels of *NLRP3*, *ASC*, *Caspase1*, *NFκB p65*, and *IκB-α*. After the addition of GA, the mRNA levels of *NLRP3*, *ASC*, *Caspase1*, *NFκB p65*, and *IκB-α* were reduced (Figure 5F). The protein expression of the NFκB/IκB-α-signaling pathway was similar to its mRNA expression. Compared with the ZEA exposure group, the group with the addition of GA significantly reduced the protein expression of NLRP3, ASC, Caspase1, p-NFκB p65, and IκB-α (*p* < 0.01) (Figure 5G–L).

### 3.6. GA Protects Cells and Reduces ZEA-Induced Cell Death

**Figure 6:**
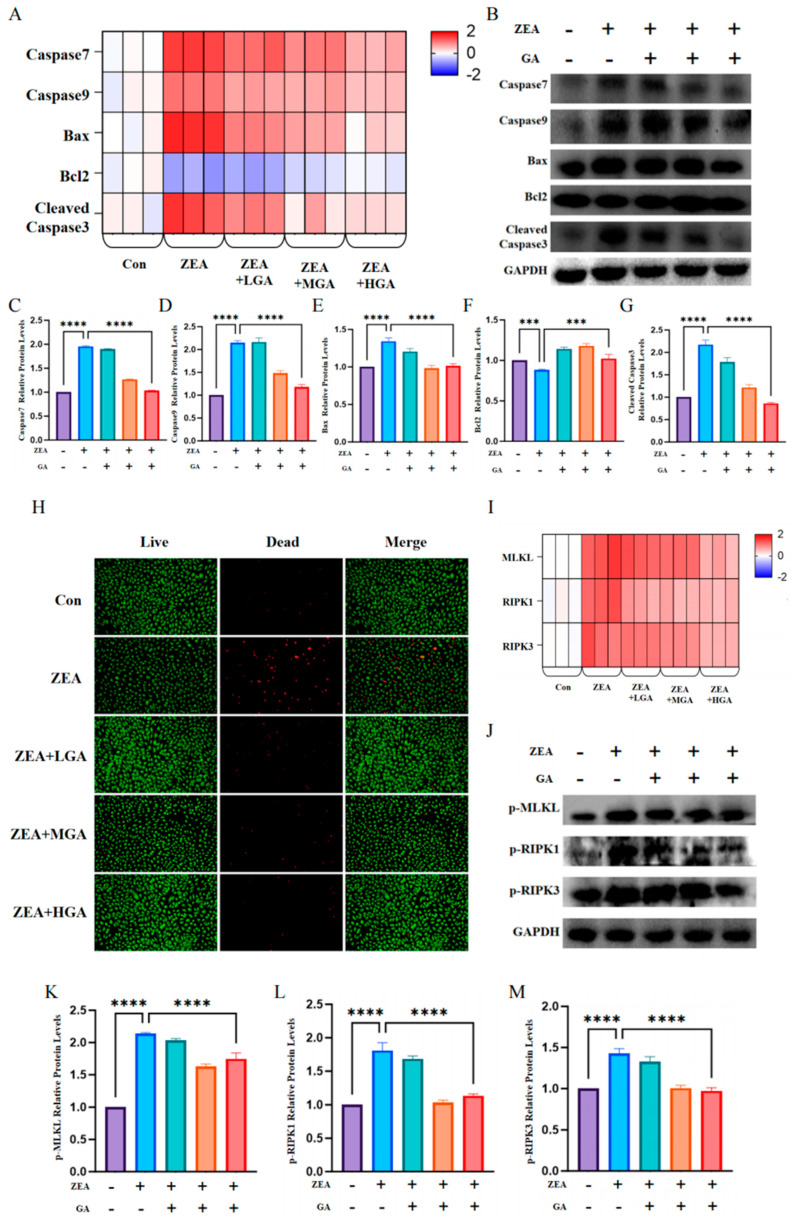
Effect of GA on ZEA−induced glandular gastric epithelial cell death in chickens. (**A**) mRNA levels of *Caspase7*, *Caspase9*, *Bax*, *Bcl2*, and *Caspase3* (*n* = 3). (**B**) Western blot images of Caspase7, Caspase9, Bax, Bcl2, and Caspase3 proteins (*n* = 10). (**C**) Protein expression of Caspase7 (*n* = 10). (**D**) Protein expression of Caspase9 (*n* = 10). (**E**) Protein expression of Bax (*n* = 10). (**F**) Protein expression of Bcl2 (*n* = 10). (**G**) Cleaved Caspase3 protein expression (*n* = 10). (**H**) A: AO/EB staining of control cells exposed to ZEA, ZEA+LGA, ZEA+MGA, and ZEA+HGA. Living cells are bright green, apoptotic cells are bright orange, and dead cells are red. (**I**) mRNA levels of *MLKL*, *RIPK1*, and *RIPK3* (*n* = 3). (**J**) Western blot images of p−MLKL, p−RIPK1, and p−RIPK3 proteins (*n* = 10). (**K**) Protein expression of p−MLKL (*n* = 10). (**L**) Expression of p−RIPK1 protein (*n* = 10). (**M**) Protein expression of p−RIPK3 (*n* = 10). *** Means with different superscripts within the same column differ (*p* < 0.005). **** Means with different superscripts within the same column differ (*p* < 0.001).

To verify that GA can reduce ZEA-induced apoptosis in chicken glandular gastric epithelial cells, we examined the expression levels of the *Caspase7*, *Caspase9*, *Bax*, *Bcl2*, and *Cleaved Caspase3* genes. The results showed that ZEA exposure significantly increased the expression of *Caspase7*, *Caspase9*, *Bax*, and *Cleaved Caspase3* and decreased the expression of the *Bcl2* gene (Figure 6A). At the same time, the expression results were verified at the protein level, and the results were identical to those obtained for their respective genes. ZEA exposure upregulated the protein levels of Caspase7, Caspase9, Bax, Bcl2, and Cleaved Caspase3. GA intervention resulted in dose-dependent decreases in the expression of the Caspase7, Caspase9, Bax, Bcl2, and Cleaved Caspase3 proteins (*p* < 0.005) (Figure 6B–G). Then, AOEB staining was performed to detect the number of live and dead cells within the cells. As shown in the figure, the number of dead cells increased significantly after ZEA exposure, and GA could effectively alleviate cell death caused by ZEA (Figure 6H). Subsequently, we tested the expression levels of the *MLKL*, *RIPK1*, and *RIPK3* genes, and the results showed that ZEA exposure significantly increased the expression levels of the *MLKL*, *RIPK1*, and *RIPK3* genes, but they also showed that, after the addition of GA, the expression levels of the *MLKL*, *RIPK1*, and *RIPK3* genes were significantly reduced (Figure 6I). To further verify the above results, a Western blot was conducted to detect the expression levels of p-MLKL, p-RIPK1, and p-RIPK3. At the protein level, the expression level of necrosis factor protein was basically consistent with the mRNA expression level (Figure 6J–M).

## 4. Discussion

ZEA is a mycotoxin that is widely found in moldy grains and can cause tissue damage in the digestive, particularly the liver, and reproductive systems. ZEA is relatively well tolerated in poultry, but long-term consumption can affect the growth performance of chicks, impair liver and kidney function, and reduce antioxidant performance [27,28]. GA has a variety of biological properties, including antioxidant, antifungal, and anti-inflammatory properties, and it has become a popular therapeutic drug [29,30]. The results show that dietary ZEA can induce inflammation of gastric gland tissue and lead to necrosis and apoptosis of gastric gland epithelial cells. GA from glycyrrhiza extract can alleviate the glandular gastric injury induced by ZEA. In this study, the glandular gastric tissue morphology in the tissue slices after adding GA to the diet tended to be normal, and the number of lesions was significantly reduced compared with that in the ZEA group. Therefore, GA may, to some extent, alleviate tissue damage caused by ZEA ingestion.

The antioxidant system plays an important role in the growth and development of birds. The content of MDA reflects the antioxidant capacity of the body and the lipid peroxide damage in tissues. ZEA exposure has been shown to significantly increase MDA content [31]. At the same time, the expression level of other antioxidant enzymes decreased, which caused oxidative stress injury to the chicken glandular stomach. Studies have confirmed that the addition of glycyrrhiza extract in feed can effectively improve the total antioxidant capacity and SOD activity of broilers [32]. Adding licorice extract to drinking water can increase the activities of GSH-PX and catalase and decrease the expression of MDA in broilers. GA is a natural antioxidant that can remove excess ROS and increase the activity of antioxidant enzymes [33]. In this study, adding different doses of GA to the feed supplemented with ZEA can significantly improve the antioxidant capacity of the chicken glandular stomach and reduce the level of MDA. In conclusion, GA can effectively reduce the damage caused by ZEA on the antioxidant function of chicken glandular stomach.

When inflammation occurs in the body, it is inevitably accompanied by the release of pro-inflammatory factors and the inhibition of anti-inflammatory factors. ZEA induces glandular gastric inflammation in chickens, increases the expression of pro-inflammatory factors (IL-6, IL-1β, and TNF-α), and inhibits the expression of anti-inflammatory factors (IL-10) [34]. Dietary ZEA supplementation can significantly increase the expression of IL-6, IL-1β, and TNF-α in glandular gastric tissue of chickens and decrease the expression of IL-10, suggesting that ZEA can promote tissue inflammation. GA reduces the binding of ZEA to organs by increasing the body’s antioxidant capacity. In this study, we found that the expression of IL-6, IL-1β, and TNF-α decreased and the expression of IL-10 increased after GA was added to ZEA-exposed diets, suggesting that GA can reduce ZEA-induced inflammation. Activation of the NFκB-signaling pathway can promote the expression of inflammatory cytokines [35]. In our study, the levels of NLRP3, ASC, Caspase1, p-NFκB, and IκB-α were significantly increased in the ZEA exposure group. During inflammation, Caspase1 is activated through autocatalytic activation [36]. The NLRP3 inflammasome is critical in host immunity to fungal, bacterial, and viral infections [37,38,39,40]. The pyrin domain of NLRP3 interacts with the pyrin domain of ASC to initiate inflammasome assembly. In addition, the NFκB-signaling pathway corresponds to NLRP3 induction [41]. The role of ROS in NLRP3 activation is still widely debated. ROS is considered to be a common signal during NLRP3 inflammasome activation [42,43]. Recent studies have shown that ROS plays a tissue-specific role in the activation of the NLRP3 inflammasome [44]. Therefore, the NFκB-signaling pathway is closely related to inflammation and apoptosis.

Inflammation and oxidative stress can induce programmed apoptosis and necrosis, and previous studies have confirmed that ZEA can also induce apoptosis and necrosis of human hepatocytes, HepaRG. Therefore, in this study, we measured and analyzed the indicators related to apoptosis and necrosis. The results showed that ZEA exposure significantly inhibited the expression of Bcl2, a classical anti-apoptotic molecule that inhibits cell death by preventing mitochondrial membrane penetration. Studies have shown that NFκB targeting Bcl2 regulates cell death, and a decrease in Bcl2 with an increase in Bax indicates an increase in cell apoptosis. In our results, ZEA exposure increased the expression level of Bax and apoptosis-related factors such as Caspase9, both confirming that ZEA induces apoptosis through the NFκB-signaling pathway. In this study, we found that ZEA can also activate the RIPK1/RIPK3/MLKL-signaling pathway to promote cell necrosis, and the in vitro results showed that the level of cell necrosis induced through exposure was significantly higher than that of apoptosis.

Our results suggest that exposure to ZEA activates the NFκB/IκB-α pathway, leading to tissue inflammation and increased expression of pro-inflammatory factors. Activation of the NFκB/IκB-α pathway decreased the expression level of Bcl2 and increased the expression level of Bax, inducing cell apoptosis. In addition, ZEA activates the RIPK1/RIPK3/MLKL-signaling pathway, inducing the occurrence of necrosis. However, the number of instances of ZEA exposure-induced cell necrosis was significantly higher than that of apoptosis, suggesting that ZEA causes tissue and cell damage primarily through cell necrosis. These results indicate that ZEA exposure can damage the glandular stomach of chickens and affect the health of poultry. Studies have shown that the body cannot completely metabolize ZEA after ingestion, and some ZEA remains in the body, causing sustained damage [45]. In recent years, research on GA has made remarkable progress regarding the treatment of inflammatory tissue damage via antioxidative stress. As a natural antioxidant, GA may become a key drug in the treatment and control of toxic diseases in chickens, so we hope that our study can provide some basis for the treatment of avian diseases with GA.

## 5. Conclusions

Our study found that ZEA exposure causes glandular gastric inflammation, apoptosis, and programmed necrosis in chickens. ZEA exposure can induce oxidative stress, significantly increase the levels of oxidative stress markers ROS and MDA, and inhibit the activity of other antioxidant enzymes. In this study, ZEA exposure induced apoptosis and necrosis of cells. The therapeutic effect of GA showed a dose-dependent pattern. These results demonstrated that GA mitigated the toxicological effects of ZEA in chicken glandular stomach and chicken glandular stomach epithelial cells. This study enriches the toxicological research on ZEA and the therapeutic effect of GA, which is of great significance for the protection of grain feed and poultry health.

## Figures and Tables

**Table 1:** Primer sequences for real-time PCR amplification.

Name	Primer Sequence Forward (5′-3′)	Primer Sequence Reverse (5′-3′)
Caspase7	AAATGAACACGGAAAACAAC	TTCTGTCTGGCTTTGCATC
Caspase9	CTCGTGGTCATCCTCTCCCAT	CCTCTCAAACTCGGGCACTGG
Bcl2	GAGTTCGGCGGCGTGATGTG	CTCGGTCATCCAGGTGGCAATG
Bax	ACTCTGCTGCTGCTCTCCTCTC	ATCCACGCAGTGCCAGATGTAATC
Caspase3	AGCAGACAGTGGACCAGATGAAAC	GGCGTGTTCCTTCAGCATCCTAC
Caspase1	GGGCTGGCAGGACTCTGATAGG	TCTCTCCCGTGGCTGGTATATGTC
NLRP3	GCTCCTTGCGTGCTCTAAGACC	TTGTGCTTCCAGATGCCGTCAG
ASC	CCAGCAAAGCCTCCGCCAAG	CAGCAATCCAGCCACCTCATACG
NFκB	GCTCACAAAGGCAGTCTCACCAG	AGGTCTCTACGCCGCTGTCAC
IκB-α	TGGCGATCATTCACGAGGAAAAGG	GTCTGGCTGAGGTTGTTCTGGAAG
RIPK1	GCCTGTTGAGAGCTGTGAGACTTC	AGGGGTGTATGGTGGAACTACTGG
RIPK3	CCCTCCATTCACTCATCCACAAGC	GCAGTGGCAGTCAGCAGGAAG
MLKL	TGCGAGCACCGATACCAGGAG	TCCAGGTCAGCCAGCAAGTCC
GAPDH	CAGAACATCATCCCAGCGTCCAC	CGGCAGGTCAGGTCAACAACAG

Caspase7, Cysteine-aspartic acid protease 7; Caspase9, Cysteine-aspartic acid protease 9; Bcl-2, B-cell lymphoma 2; Bax, Bcl-2-associated X Protein; Caspase3, Cysteine-aspartic acid protease 3; Caspase1, Cysteine-aspartic acid protease 1; NLRP3, Nucleotide-binding oligomerization domain-like receptor protein 3; ASC, Apoptosis-associated speck-like protein containing a CARD; NFκB, Nuclear Factor kappa-light-chain-enhancer of activated B cells; IκB-α, Inhibitor of kappa B-α; RIPK1, Receptor-Interacting Protein Kinase 3; RIPK1, Receptor-Interacting Protein Kinase 3; MLKL, Mixed Lineage Kinase Domain-Like; GAPDH, Glyceraldehyde-3-phosphate dehydrogenase; F, forward; R, reverse.

## Data Availability

The original contributions presented in the study are included in the article, further inquiries can be directed to the corresponding author.

## References

[1] Ropejko K., Twarużek M. (2021). Zearalenone and Its Metabolites-General Overview, Occurrence, and Toxicity. Toxins.

[2] Schothorst R.C., van Egmond H.P. (2004). Report from SCOOP task 3.2.10 “collection of occurrence data of Fusarium toxins in food and assessment of dietary intake by the population of EU member states”. Subtask: Trichothecenes. Toxicol. Lett..

[3] Han X., Huangfu B., Xu T., Xu W., Asakiya C., Huang K., He X. (2022). Research Progress of Safety of Zearalenone: A Review. Toxins.

[4] Raj J., Farkaš H., Jakovčević Z., Medina A., Magan N., Čepela R., Vasiljević M. (2022). Comparison of multiple mycotoxins in harvested maize samples in three years (2018-2020) in four continents. Food Addit. Contam. Part A.

[5] Zhu F., Zhu L., Xu J., Wang Y., Wang Y. (2023). Effects of moldy corn on the performance, antioxidant capacity, immune function, metabolism and residues of mycotoxins in eggs, muscle, and edible viscera of laying hens. Poult. Sci..

[6] Bi Z., Gu X., Xiao Y., Zhou Y., Bao W., Wu S., Wang H. (2022). Analysis of the Roles of the ISLR2 Gene in Regulating the Toxicity of Zearalenone Exposure in Porcine Intestinal Epithelial Cells. Toxins.

[7] Shen T., Miao Y., Ding C., Fan W., Liu S., Lv Y., Gao X., De Boevre M., Yan L., Okoth S. (2019). Activation of the p38/MAPK pathway regulates autophagy in response to the CYPOR-dependent oxidative stress induced by zearalenone in porcine intestinal epithelial cells. Food Chem. Toxicol..

[8] (2013). Knapp BK, Bauer LL, Swanson KS, Tappenden KA, Fahey GC Jr, de Godoy MR. Soluble fiber dextrin and soluble corn fiber supplementation modify indices of health in cecum and colon of Sprague-Dawley rats. Nutrients.

[9] Debevere S., Cools A., De Baere S., Haesaert G., Rychlik M., Croubels S., Fievez V. (2020). In Vitro Rumen Simulations Show a Reduced Disappearance of Deoxynivalenol, Nivalenol and Enniatin B at Conditions of Rumen Acidosis and Lower Microbial Activity. Toxins.

[10] Kemboi D.C., Antonissen G., Ochieng P.E., Croubels S., Okoth S., Kangethe E.K., Faas J., Lindahl J.F., Gathumbi J.K. (2020). A Review of the Impact of Mycotoxins on Dairy Cattle Health: Challenges for Food Safety and Dairy Production in Sub-Saharan Africa. Toxins.

[11] Tardieu D., Travel A., Le Bourhis C., Metayer J.-P., Mika A., Cleva D., Boissieu C., Guerre P. (2021). Fumonisins and zearalenone fed at low levels can persist several days in the liver of turkeys and broiler chickens after exposure to the contaminated diet was stopped. Food Chem. Toxicol..

[12] Fu Y., Jin Y., Tian Y., Yu H., Wang R., Qi H., Feng B., Zhang J. (2022). Zearalenone Promotes LPS-Induced Oxidative Stress, Endoplasmic Reticulum Stress, and Accelerates Bovine Mammary Epithelial Cell Apoptosis. Int. J. Mol. Sci..

[13] Yi Y., Gao K., Zhang L., Lin P., Wang A., Jin Y. (2022). Zearalenone Induces MLKL-Dependent Necroptosis in Goat Endometrial Stromal Cells via the Calcium Overload/ROS Pathway. Int. J. Mol. Sci..

[14] Zhang P., Jing C., Liang M., Jiang S., Huang L., Jiao N., Li Y., Yang W. (2021). Zearalenone Exposure Triggered Cecal Physical Barrier Injury through the TGF-β1/Smads Signaling Pathway in Weaned Piglets. Toxins.

[15] Fan W., Shen T., Ding Q., Lv Y., Li L., Huang K., Yan L., Song S. (2017). Zearalenone induces ROS-mediated mitochondrial damage in porcine IPEC-J2 cells. J. Biochem. Mol. Toxicol..

[16] Guijarro-Muñoz I., Compte M., Álvarez-Cienfuegos A., Álvarez-Vallina L., Sanz L. (2014). Lipopolysaccharide activates Toll-like receptor 4 (TLR4)-mediated NF-κB signaling pathway and proinflammatory response in human pericytes. J. Biol. Chem..

[17] Pistol G.C., Gras M.A., Marin D.E., Israel-Roming F., Stancu M., Taranu I. (2014). Natural feed contaminant zearalenone decreases the expressions of important pro- and anti-inflammatory mediators and mitogen-activated protein kinase/NF-κB signalling molecules in pigs. Br. J. Nutr..

[18] Sun L., He D., Liu Y., Wei Y., Wang L. (2023). Corynoline protects against zearalenone-induced liver injury by activating the SIRT1/Nrf2 signaling pathway. J. Biochem. Mol. Toxicol..

[19] Xu J., Li S., Jiang L., Gao X., Liu W., Zhu X., Huang W., Zhao H., Wei Z., Wang K. (2021). Baicalin protects against zearalenone-induced chicks liver and kidney injury by inhibiting expression of oxidative stress, inflammatory cytokines and caspase signaling pathway. Int. Immunopharmacol..

[20] Luo J.-J., Zhang Y., Sun H., Wei J.-T., Khalil M.M., Wang Y.-W., Dai J.-F., Zhang N.-Y., Qi D.-S., Sun L.-H. (2019). The response of glandular gastric transcriptome to T-2 toxin in chicks. Food Chem. Toxicol..

[21] Heidari S., Mehri S., Hosseinzadeh H. (2021). The genus Glycyrrhiza (Fabaceae family) and its active constituents as protective agents against natural or chemical toxicities. Phytother. Res..

[22] Sharifi-Rad J., Quispe C., Herrera-Bravo J., Belén L.H., Kaur R., Kregiel D., Uprety Y., Beyatli A., Yeskaliyeva B., Kırkın C. (2021). Glycyrrhiza Genus: Enlightening Phytochemical Components for Pharmacological and Health-Promoting Abilities. Oxid. Med. Cell Longev..

[23] Xu R., Han F.X., Wang H.R., Wang J.J., Cai Z.L., Guo M.Y. (2024). Tea polyphenols alleviate TBBPA-induced inflammation, ferroptosis and apoptosis via TLR4/NF-κB pathway in carp gills. Fish. Shellfish. Immunol..

[24] Cui J., Zhu M., Sun X., Yang J., Guo M. (2024). Microplastics induced endoplasmic reticulum stress to format an inflammation and cell death in hepatocytes of carp (Cyprinus carpio). Aquat. Toxicol..

[25] Wu J., Zhang Y., Liu T., Yang J., Sun X., Gao X.J. (2024). The mechanism of selenium regulating the permeability of vascular endothelial cells through selenoprotein O. Redox Biol..

[26] Zhang Q., Wang F., Xu S., Cui J., Li K., Shiwen X., Guo M.-Y. (2023). Polystyrene microplastics induce myocardial inflammation and cell death via the TLR4/NF-κB pathway in carp. Fish Shellfish. Immunol..

[27] Jia S., Ren C., Yang P., Qi D. (2022). Effects of Intestinal Microorganisms on Metabolism and Toxicity Mitigation of Zearalenone in Broilers. Animals.

[28] Król A., Pomastowski P., Rafińska K., Railean-Plugaru V., Walczak J., Buszewski B. (2018). Microbiology neutralization of zearalenone using Lactococcus lactis and *Bifidobacterium* sp. Anal. Bioanal. Chem..

[29] Xu C., Liang C., Sun W., Chen J., Chen X. (2018). Glycyrrhizic acid ameliorates myocardial ischemic injury by the regulation of inflammation and oxidative state. Drug Des. Dev. Ther..

[30] Wang H., Ge X., Qu H., Wang N., Zhou J., Xu W., Xie J., Zhou Y., Shi L., Qin Z. (2020). Glycyrrhizic Acid Inhibits Proliferation of Gastric Cancer Cells by Inducing Cell Cycle Arrest and Apoptosis. Cancer Manag. Res..

[31] Xia S., Yan C., Gu J., Yuan Y., Zou H., Liu Z., Bian J. (2024). Resveratrol Alleviates Zearalenone-Induced Intestinal Dysfunction in Mice through the NF-κB/Nrf2/HO-1 Signalling Pathway. Foods.

[32] Chen Y., Cheng Y., Wen C., Wang W., Kang Y., Wang A., Zhou Y. (2019). The protective effects of modified palygorskite on the broilers fed a purified zearalenone-contaminated diet. Poult. Sci..

[33] Ojha S., Javed H., Azimullah S., Abul Khair S.B., Haque M.E. (2016). Glycyrrhizic acid Attenuates Neuroinflammation and Oxidative Stress in Rotenone Model of Parkinson’s Disease. Neurotox Res..

[34] Lee P.-Y., Liu C.-C., Wang S.-C., Chen K.-Y., Lin T.-C., Liu P.-L., Chiu C.-C., Chen I.-C., Lai Y.-H., Cheng W.-C. (2021). Mycotoxin Zearalenone Attenuates Innate Immune Responses and Suppresses NLRP3 Inflammasome Activation in LPS-Activated Macrophages. Toxins.

[35] Guerrero-Hue M., García-Caballero C., Palomino-Antolín A., Rubio-Navarro A., Vázquez-Carballo C., Herencia C., Martín-Sanchez D., Farré-Alins V., Egea J., Cannata P. (2019). Curcumin reduces renal damage associated with rhabdomyolysis by decreasing ferroptosis-mediated cell death. FASEB J..

[36] Manji G.A., Wang L., Geddes B.J., Brown M., Merriam S., Al-Garawi A., Mak S., Lora J.M., Briskin M., Jurman M. (2002). PYPAF1, a PYRIN-containing Apaf1-like protein that assembles with ASC and regulates activation of NF-kappa B. J. Biol. Chem..

[37] Thomas P.G., Dash P., Aldridge J.R., Ellebedy A.H., Reynolds C., Funk A.J., Martin W.J., Lamkanfi M., Webby R.J., Boyd K.L. (2009). The intracellular sensor NLRP3 mediates key innate and healing responses to influenza A virus via the regulation of caspase-1. Immunity.

[38] Allen I.C., Scull M.A., Moore C.B., Holl E.K., McElvania-TeKippe E., Taxman D.J., Guthrie E.H., Pickles R.J., Ting J.P.-Y. (2009). The NLRP3 inflammasome mediates in vivo innate immunity to influenza A virus through recognition of viral RNA. Immunity.

[39] Gross O., Poeck H., Bscheider M., Dostert C., Hannesschläger N., Endres S., Hartmann G., Tardivel A., Schweighoffer E., Tybulewicz V. (2009). Syk kinase signalling couples to the Nlrp3 inflammasome for anti-fungal host defence. Nature.

[40] Kanneganti T.-D., Body-Malapel M., Amer A., Park J.-H., Whitfield J., Franchi L., Taraporewala Z.F., Miller D., Patton J.T., Inohara N. (2006). Critical role for Cryopyrin/Nalp3 in activation of caspase-1 in response to viral infection and double-stranded RNA. J. Biol. Chem..

[41] Bauernfeind F.G., Horvath G., Stutz A., Alnemri E.S., MacDonald K., Speert D., Fernandes-Alnemri T., Wu J., Monks B.G., Fitzgerald K.A. (2009). Cutting edge: NF-kappaB activating pattern recognition and cytokine receptors license NLRP3 inflammasome activation by regulating NLRP3 expression. J. Immunol..

[42] Dostert C., Pétrilli V., Van Bruggen R., Steele C., Mossman B.T., Tschopp J. (2008). Innate immune activation through Nalp3 inflammasome sensing of asbestos and silica. Science.

[43] Cruz C.M., Rinna A., Forman H.J., Ventura A.L., Persechini P.M., Ojcius D.M. (2007). ATP activates a reactive oxygen species-dependent oxidative stress response and secretion of proinflammatory cytokines in macrophages. J. Biol. Chem..

[44] Ma M.W., Wang J., Dhandapani K.M., Brann D.W. (2017). NADPH Oxidase 2 Regulates NLRP3 Inflammasome Activation in the Brain after Traumatic Brain Injury. Oxid. Med. Cell. Longev..

[45] Bao L., Huang Y., Gu F., Liu W., Guo Y., Chen H., Wang K., Wu Z., Li J. (2024). Zearalenone induces liver injury in mice through ferroptosis pathway. Sci. Total Environ..

